# Prediction of (Non)Participation of Older People in Digital Health Research: Exergame Intervention Study

**DOI:** 10.2196/17884

**Published:** 2020-06-05

**Authors:** Arianna Poli, Susanne Kelfve, Leonie Klompstra, Anna Strömberg, Tiny Jaarsma, Andreas Motel-Klingebiel

**Affiliations:** 1 Division Ageing and Social Change Department of Culture and Society Linköping University Norrköping Sweden; 2 Aging Research Center Karolinska Institutet and Stockholm University Stockholm Sweden; 3 Department of Health, Medicine and Caring Science Linköping University Linköping Sweden; 4 Department of Cardiology Linköping University Linköping Sweden; 5 Julius Centre University Medical Center Utrecht Netherlands

**Keywords:** technology, exclusion, recruitment, self-selection, nonparticipation

## Abstract

**Background:**

The use of digital technologies is increasing in health care. However, studies evaluating digital health technologies can be characterized by selective nonparticipation of older people, although older people represent one of the main user groups of health care.

**Objective:**

We examined whether and how participation in an exergame intervention study was associated with age, gender, and heart failure (HF) symptom severity.

**Methods:**

A subset of data from the HF-Wii study was used. The data came from patients with HF in institutional settings in Germany, Italy, the Netherlands, and Sweden. Selective nonparticipation was examined as resulting from two processes: (non)recruitment and self-selection. Baseline information on age, gender, and New York Heart Association Functional Classification of 1632 patients with HF were the predictor variables. These patients were screened for HF-Wii study participation. Reasons for nonparticipation were evaluated.

**Results:**

Of the 1632 screened patients, 71% did not participate. The nonrecruitment rate was 21%, and based on the eligible sample, the refusal rate was 61%. Higher age was associated with lower probability of participation; it increased both the probabilities of not being recruited and declining to participate. More severe symptoms increased the likelihood of nonrecruitment. Gender had no effect. The most common reasons for nonrecruitment and self-selection were related to physical limitations and lack of time, respectively.

**Conclusions:**

Results indicate that selective nonparticipation takes place in digital health research and that it is associated with age and symptom severity. Gender effects cannot be proven. Such systematic selection can lead to biased research results that inappropriately inform research, policy, and practice.

**Trial Registration:**

ClinicalTrial.gov NCT01785121, https://clinicaltrials.gov/ct2/show/NCT01785121

## Introduction

Increasingly more digital health technologies are evaluated for their potential use in the provision of health care services, with promising results at both individual and organizational levels [1-3]. It is expected that current health care service provision will be progressively restructured around the use of digital health technologies [4,5]. The use of digital technologies in health care service provision involves all users and hence, older people, as they represent one of the main user groups of health care services [6].

Although the number of older people using digital technologies is increasing [7], many are either nonusers or have little experience with using new technologies [8,9], even in countries that show high levels of internet and digital technology use [10].

Differences in the use of digital technologies exist not only between younger and older people but also within the older population [9]. Lower use and nonuse of digital technologies in old age are generally related to material, social, and individual resources [11]. Previous studies have found that lower education levels, less income, and poorer health status are associated with less use or nonuse of digital technologies [12-16]. Limited use or nonuse of digital technologies can also be determined by individual choices [17].

Such digital disparities can lead to disadvantages for some groups and hence contribute to increased risks of social exclusion and widened social inequality. Disparities can be reinforced by the participant selection strategies in studies evaluating new digital technologies [1,18,19]. Evaluations of digital health technologies typically follow the laboratory phases in which the digital technologies are developed and customized and focus on evaluating such tools with future target users via pilot trials or randomized trials [2]. However, such evaluations have some important shortcomings. Among others, the selection of study participants often results in samples that do not reflect the target population of interest [18,19] and hence, predetermines selective nonparticipation in the studies.

Selective nonparticipation in research prevents acknowledging, representing, and recognizing the interests and needs of different people [20,21], leading to biased research results [18,22] and generation of recommendations that are inaccurate for the entire population of interest. Underrepresented groups might be excluded or benefit less from the opportunities provided by the use of digital technologies in health care service provision and, most importantly, be disadvantaged by this change, compared with their digitally engaged counterparts. Such a disadvantage holds the potential to widen existing old-age inequalities [23,24].

Previous research has found that people who participate in studies on digital health technologies are typically younger, with better subjective health and overall quality of life; are more often men; are in better socioeconomic conditions; report more frequent digital access; and have higher digital skills [25-29]. Merkel et al [18] emphasized that vulnerable populations are less likely to be involved in such studies. In addition, refusal rates are often quite high [25,29,30]. Overall, people who agree to participate in studies can differ significantly from those who decline [29].

There are few studies that describe processes that result in selective nonparticipation of older people in digital health research [26,27,29]. Previous studies have mainly focused on individual decision making regarding whether to participate. However, less is known about selective nonparticipation resulting from the combination of two processes: (non)recruitment according to study requirements and self-selection as an individual decision.

The aim of this paper was to understand what predicts (non)participation of older people in digital health research. Here, participation is defined as being involved in digital health research as a study participant, and nonparticipation is defined as not being involved in digital health research. To study this, we conducted an analysis that deconstructs and examines (non)recruitment and self-selection occurring in a study evaluating exergame technology for patients with heart failure (HF); detailed descriptions of both the study and the intervention have been reported previously [31,32]. We investigated whether patients who were not eligible, those who declined, and those who accepted participation differed according to age, gender, and HF symptom severity. We hypothesized that age, gender, and symptom severity predicted individual participation in the HF-Wii study. We expected that participants would be younger, more likely to be men, and have less severe symptoms compared with those who were not eligible and those who declined to participate.

## Methods

### HF-Wii Study

This study was conducted within the research program “Supporting Self-Care by Information and Communication Technology for Older People with Long-Term Conditions” (ICT4Self-care; 2015-2018) conducted at Linköping University and funded by the Swedish Research Council for Health, Working Life and Welfare (FORTE; dnr 2014-4100). The analyses are based on data collected from the HF-Wii study [31,33], which contributed to this research program.

The HF-Wii study evaluated the impact of exergaming on the exercise capacity and daily physical activity of patients with HF. Exergame is a term that refers to video games that can be used for exercising, often at home. The HF-Wii study is based on a randomized controlled trial (RCT) approach. The HF-Wii study was developed based on the results obtained from a case study and a pilot study in which patients with HF were involved. Based on the experiences gained from these studies (ie, evaluating the results, experiences of patients with an exergame platform, and experiences of the research staff in conducting an exergame study), the RCT was developed [33,34]. Furthermore, a research partner (a patient with HF) from the Swedish Heart and Lung Foundation was involved in refining the instruction session, questionnaires, and recruitment and data collection strategies.

The target population of the HF-Wii study consisted of patients who were diagnosed with HF by a cardiologist according to European Society of Cardiology guidelines [35] and who were older than 18 years. Exclusion criteria in the HF-Wii study were physical or balance problems, visual impairments, severe cognitive impairment(s) or psychiatric illness, a life expectancy shorter than 6 months, and not being able to speak or understand the language of the country where the study took place. Such criteria were assessed by a recruiter in each of the countries. Patients who were eligible according to the criteria were invited to participate in the HF-Wii study.

This study is based on data that refer to the study inclusion from four of the countries involved in the HF-Wii study: Germany, Italy, the Netherlands, and Sweden. Ethical approval for the HF-Wii study was obtained from local ethical committees (in Germany, GERS22(a)/2015; in Italy, IT:0052838/272/UVF/1; in the Netherlands, NL48647.068.14/METC141085; in Sweden, DNR 2012/247-31).

### Data

The study population for this research in the four countries (Germany, Italy, the Netherlands, and Sweden) consisted of 1632 patients with HF who were screened in institutional settings between 2013 and 2017 for participation in the HF-Wii study. For these patients with HF, baseline information on age, gender, and HF symptom severity was completed by the recruiters in the four countries. Information on age was available for 1567 patients. Data on gender were retrieved for 1552 patients, and New York Heart Association (NYHA) class was recorded for 1180 patients.

Furthermore, data on (non)participation in the HF-Wii study based on the recruitment logs and reasons for nonparticipation were collected. No personal nor sensitive data were collected for this analysis.

### Selective Nonparticipation

Selective nonparticipation was studied as resulting from two succeeding processes in the recruitment phase of the HF-Wii study: (non)recruitment according to the study requirements and self-selection as an individual decision [36]. The (non)recruitment process refers to the initial screening to select who is eligible to participate and excludes the others (ie, nonrecruited group). In contrast, self-selection is based on individual decision making that results in accepting or declining the invitation to participate and distinguishes between participants and decliners.

### Outcome Variable

The outcome variable was constructed as a categorical variable with 3 categories, namely the 3 groups in which patients could alternatively be classified as a result of the recruitment phase: nonrecruited, decliner, or participant. The categories were constructed based on information from the recruitment logs that reported whether a patient was not recruited (ie, ineligible because of exclusion criteria), a decliner (ie, the patient declined the invitation to participate in the study), or a participant in the HF-Wii study.

### Predictor Variables

Age, gender, and HF symptom severity were the predictor variables of this study. Age was included as a categorical variable: ≤64 years, 65-69 years, 70-79 years, ≥80 years, or missing. Gender was categorized as male, female, or missing. HF symptom severity was assessed by HF nurses according to the NYHA Functional Classification [37]. This classification is based on a subjective assessment of symptoms ranging between class I (ie, no symptoms and no limitation in ordinary physical activity, but presence of shortness of breath when walking or climbing stairs) and class IV (ie, severe limitations, experience of symptoms even while at rest). For the analyses, a missing value category was used.

Documented reasons for nonrecruitment and self-selection may further explain (non)recruitment and self-selection. Therefore, for those patients who were not eligible based on the HF-Wii study criteria, reasons for nonrecruitment were documented. For those patients who did not want to participate, reasons for declining the invitation were collected as free-text responses and coded into 10 categories: not having time, working or travelling a lot, unwilling to come to follow-up meetings, having other illnesses, already exercising a lot, unwilling to use technical equipment or the exergame device, already have a exergame device, living between different houses (unwillingness to move the exergame equipment from place to place), and shared living. Reasons for declining that did not fall into any of the above listed 10 categories were classified as “other.”

### Analyses

First, descriptive analyses were performed to describe the predictor variables in the 3 groups and to illustrate nonrecruitment, declining, and participation rates. One-way ANOVA and Pearson’s chi-squared test were used for testing differences among the 3 groups.

Second, multinomial logistic regression was used to test the association between recruitment group membership (ie, nonrecruited, decliner, participant) and the predictors age, gender, and symptom severity. The participant group was the reference category.

Based on the regression model, we calculated the average marginal effects (AMEs) for each of the categories of the predictor variables. For each category of the predictor variables in the model, the AME showed the probability of being part of the nonrecruited, decliner, or participant groups for an individual who has the same values on every independent variable in the model except one [38]. AMEs were used because they could be compared more easily than odds ratios across groups in the sample [39].

In these analyses, we did not include patients for whom information on age and gender was missing. Inclusion of patients with missing data in the analyses was checked but did not result in a significant improvement of the model. However, we kept the missing value category for the variable NYHA as it concerns a larger number of cases. The sample size for these analyses consisted of 1489 patients (ie, net sample).

Third, to illustrate the selection processes, the reasons why patients were deemed ineligible, and why they decided not to participate in the HF-Wii study were evaluated. Reasons for ineligibility were investigated for all patients in the nonrecruited group, and reasons for individuals not to participate were described for patients in the decliner group.

Analyses were performed using SPSS Statistics version 25.0 (IBM Corp, Armonk, NY) and Stata software version 15 (StataCorp, College Station, TX).

## Results

### Patient Characteristics

Overall, 1632 patients with HF entered the recruitment phase of the HF-Wii study in the four countries. The mean age of the patients was 70 years (SD 11.9 years). More than half of the patients who entered the recruitment phase were ≥70 years old, representing 55.58% (907/1632) of the sample, and 13.30% (217/1632) of the patients were between 65 and 69 years of age. Around one quarter of the sample (443/1632, 27.14%) was ≤64 years old. No information on age was available for the remaining 3.98% (65/1632) of the patients. Of the 1632 patients screened, 64.83% (1058/1632) were men, and 30.27% (494/1632) were women. Information was missing for 4.90% (80/1632) of patients.

Of all the patients, 35.66% (582/1632) were classified as having mild HF symptoms and somewhat limited ability to exercise (NYHA class II), and 27.76% (453/1632) had marked limitations in activity due to symptoms (NYHA class III). Only 2.39% (39/1632) of patients had severe HF symptoms even at rest (NYHA class IV), and 6.50% (106/1632) had no HF symptoms (NYHA class I). Information on HF symptom severity was missing for 452 patients (452/1632, 27.70%).

### Nonrecruitment, Declining, and Participation Rates

Overall, 71.45% (1166/1632) of all patients screened for the HF-Wii study did not participate in the intervention study (Table 1). Among those who did not participate, 37.99% (443/1166) were nonrecruited, and 62.01% (723/11166) declined to participate. The refusal rate for the HF-Wii study, based only on those patients who were invited to the study, was 60.81% (723/1189).

Mean ages were significantly different between the nonrecruited (73 years, SD 12.2 years), decliner (70 years, SD 11.8 years), and participant (67 years, SD 11.2 years) groups (*F*_2,1566_=29.2, *P*<.001). Participants were significantly younger than patients in the decliner and nonrecruited groups.

Among the different age groups, the participation rate was lowest for patients 80 years old and older (59/361, 16.3%). The highest participation rate was found among patients 65-69 years of age (89/217, 41.0%). On the other hand, it was more common for patients 80 years old and older to be nonrecruited (155/361, 42.9%), compared with all the other groups (70-79 years, 127/546, 23.3%; 65-69 years, 40/217, 18.4%; and ≤64 years, 97/443, 21.9%). Declining to participate in the RCT was more common among patients 70-79 years of age (257/546, 47.1%), compared with the other age groups, for which declining varied from 40.6% to 42.9%.

On average, it was more common for men to participate in the HF-Wii study (332/1058, 30.38%) compared with women (134/494, 27.1%). In contrast, women were more often nonrecruited (168/494, 34.0%) than men (259/1058, 24.48%), while men more often declined participation (467/1058, 44.14%) compared with women (192/494, 38.9%). No information on gender was available for 16 patients in the nonrecruited group and for 64 patients in the decliner group.

On average, participation was lower among patients with marked limitations in activity due to HF symptoms and severe HF symptoms at rest (NYHA class III, 105/453, 23.2%; NYHA class IV, 4/39, 10.3%) compared with patients with no HF symptoms (NYHA class I, 55/106, 51.9%) or mild HF symptoms (NYHA class II, 280/582, 48.1%). Being ineligible for the HF-Wii study was more common among patients with NYHA class IV HF (28/39, 71.8%) than among the other groups for which the rate of nonrecruitment varied from 4.7% to 44.6%.

**Table 1:** Characteristics of the 1632 patients, by recruitment group.

Characteristics		Recruitment groups			Statistic	*P* value
		Nonrecruited, n (%)	Decliner, n (%)	Participant, n (%)		
Group size		443 (27.14)	723 (44.30)	466 (28.55)		
**Age range (years)**						
	≤64	97 (21.90)	190 (42.89)	156 (35.21)	χ^2^_8_=113.4	<.001
	65-69	40 (18.43)	88 (40.55)	89 (41.01)		
	70-79	127 (23.26)	257 (47.07)	162 (29.67)		
	≥80	155 (42.94)	147 (40.72)	59 (16.34)		
	Missing	24 (36.92)	41 (63.08)	0 (0)		
Age (years)		73 (12.2)^a^	70 (11.8)^a^	67 (11.2)^a^	*F*_2,1556_=29.2	<.001
**Gender**						
	Men	259 (24.48)	467 (44.14)	332 (31.38)	χ^2^_2_=65.3	<.001
	Women	168 (34.01)	192 (38.87)	134 (27.13)		
	Missing	16 (20.00)	64 (80.00)	0 (0)		
**HF^b^ symptom severity**						
	NYHA class I^c^	5 (4.72)	46 (43.40)	55 (51.89)	χ^2^_8_=416.6	<.001
	NYHA class II^d^	60 (10.31)	242 (41.58)	280 (48.11)		
	NYHA class III^e^	202 (44.59)	146 (32.23)	105 (23.18)		
	NYHA class IV^f^	28 (71.79)	7 (17.95)	4 (10.26)		
	Missing	148 (32.74)	282 (62.39)	22 (4.87)		

^a^mean (SD).

^b^HF: heart failure.

^c^NYHA class I: no limitation of physical activity. Ordinary physical activity does not cause undue fatigue, palpitation, dyspnea (shortness of breath) [37].

^d^NYHA class II: slight limitation of physical activity. Comfortable at rest. Ordinary physical activity results in fatigue, palpitation, dyspnea (shortness of breath) [37].

^e^NYHA class III: marked limitation of physical activity. Comfortable at rest. Less than ordinary activity causes fatigue, palpitation, or dyspnea [37].

^f^NYHA class IV: unable to carry on any physical activity without discomfort. Symptoms of HF at rest. If any physical activity is undertaken, discomfort increases [37].

### Participation in the HF-Wii Study

A multinomial logistic regression was performed to test the association between recruitment group membership (ie, nonrecruited, decliner, participant) and the predictors age, gender, and HF symptom severity. The reference group consisted of patients who were recruited and agreed to participate (ie, participant group; Table 2).

**Figure 1:**
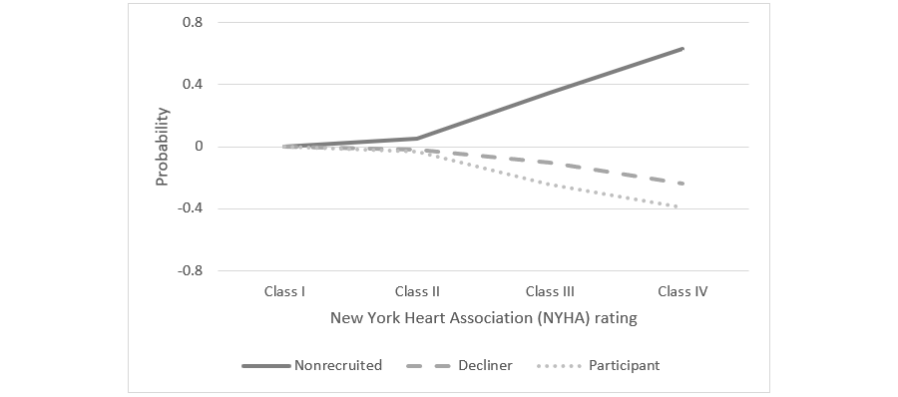
Probability of being in one of the 3 recruitment groups (ie, nonrecruited, decliner, participant) based on heart failure symptom severity.

The model shows that HF symptom severity, according to the NYHA functional classification, significantly predicted the likelihood of participation in the HF-Wii study. Patients with more severe symptoms, namely those in NYHA classes III and IV, had a decreased probability of participating and an increased probability of not being recruited, compared with patients with no or mild symptoms (ie, those in NYHA classes I and II). When compared with patients who displayed no symptoms (ie, NYHA class I), patients who had more severe HF symptoms (ie, NYHA classes III and IV) were significantly more likely to be nonrecruited to the HF-Wii study than to participate in it (NYHA class III, odds ratio [OR] 14.68, *P*<.001; NYHA class IV, OR 56.18, *P*<.001). Average marginal effects showed that more severe HF symptoms increased the probability of not being recruited by 63 percentage points for patients with NYHA class IV and by 35 percentage points for patients with NYHA class III (Figure 1). More severe HF symptoms decreased the probability of participation by 3 percentage points for patients with NYHA class I, by 25 percentage points for patients with NYHA class II, and by 39 percentage points for patients with NYHA class III.

**Table 2:** Multinomial logistic regression model of the relationship between recruitment group membership and predictors (ie, age, gender, and heart failure [HF] symptom severity) for all the patients for whom information on age and gender was available (n=1489) in the HF-Wii study. Pseudo *R*^2^ (McFadden)=0.13.

Predictors		Nonrecruited vs participant^a^				Decliner vs participant^a^					Non recruited		Decliner		Participant	
		β	SE	*P* value	Exp(β)		β	SE	*P* value	Exp(β)		AME^b^ (SD)		AME (SD)		AME (SD)
Intercept		–2.93	.51	.00	N/A^c^		–0.64	.26	.01	N/A		N/A		N/A		N/A
**Age range (years)**																
	≤64	.57	.26	.03	1.78		.29	.20	.15	1.34		.06 (.04)		.01 (.03)		–.08 (.04)
	65-69 (ref.)	0	N/A	N/A	N/A		0	N/A	N/A	N/A		0		0		0
	70-79	.46	.25	.07	1.59		.55	.20	.01	1.74		.02 (.03)		.08 (.04)		–.10 (.04)
	≥80	1.35	.27	<.001	3.86		.89	.24	<.001	2.44		.13 (.04)		.06 (.04)		–.19 (.04)
**Gender**																
	Women	.19	.16	.24	1.21		–.04	.14	.77	.96		.04 (.02)		–.03 (.03)		–.01 (.02)
	Men (ref)	0	N/A	N/A	N/A		0	N/A	N/A	N/A		0		0		0
**HF symptom severity^d^**																
	NYHA class I^e^ (ref)	0	N/A	N/A	N/A		0	N/A	N/A	N/A		0		0		0
	NYHA class II^f^	.70	.49	.15	2.02		.03	.23	.91	1.03		.05 (.03)		–.02 (.05)		–.03 (.05)
	NYHA class III^g^	2.69	.49	<.001	14.68		.41	.25	.11	1.51		.35 (.04)		–.10 (.06)		–.25 (.06)
	NYHA class IV^h^	4.03	.72	<.001	56.18		.67	.67	.32	1.95		.63 (.08)		–.24 (.08)		–.39 (.08)

^a^Reference category.

^b^AME: average marginal effect.

^c^N/A: not applicable.

^d^The missing value category was included in the analyses but is not reported in this table.

^e^NYHA class I: no limitation of physical activity. Ordinary physical activity does not cause undue fatigue, palpitation, dyspnea (shortness of breath) [37].

^f^NYHA class II: slight limitation of physical activity. Comfortable at rest. Ordinary physical activity results in fatigue, palpitation, dyspnea (shortness of breath) [37].

^g^NYHA class III: marked limitation of physical activity. Comfortable at rest. Less than ordinary activity causes fatigue, palpitation, or dyspnea [37].

^h^NYHA class IV: unable to carry on any physical activity without discomfort. Symptoms of HF at rest. If any physical activity is undertaken, discomfort increases [37].

**Figure 2:**
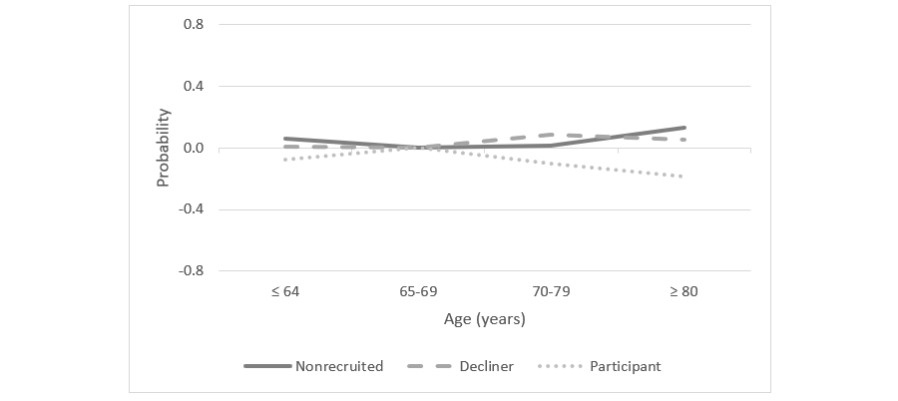
Probability of being in one of the 3 recruitment groups (ie, nonrecruited, decliner, participant) based on age.

The model also reveals that age was a significant predictor for participation in the HF-Wii study and contributed to explaining both (non)recruitment based on study requirements and self-selection as an individual decision. Being older reduced the probability of participating and of agreeing to participate. Compared with those 65-69 years of age, patients 80 years old and older were more likely to not be recruited than to be participants in the HF-Wii study (OR 3.86, *P*<.001). Probability of being not recruited increased by 13 percentage points for patients 80 years old and older, when compared with those 65-69 years of age (Figure 2). This was also found among patients 64 years of age and younger (OR 1.78, *P=*.03).

Compared with the patients 65-69 years of age, people in the two older age groups (ie, 70-79 years and ≥80 years) were significantly more likely to decline the invitation to participate in the HF-Wii study than to participate in it (OR 1.74, *P*=.01; OR 2.44, *P*<.001, respectively). Being older increased the probability of declining participation in the study by 8 percentage points for patients 70-79 years of age and by 6 percentage points for those who were 80 years old and older, compared with patients 65-69 years of age.

Gender was not associated with the likelihood of participation in the HF-Wii study. It had no effect on the probability of being in the nonrecruited, decliner, or participant groups.

### Reasons for Non-Recruitment and Declining to Participate

Of the patients, 27.14% (443/1632) were nonrecruited and thus were not invited to participate in the HF-Wii study. Patients were nonrecruited if they met at least one of the exclusion criteria.

The two most common reasons for being nonrecruited in the HF-Wii study were balance (223/443) and other physical problems (189/443; Table 3). Both reasons were more common among patients with marked and severe HF symptoms (ie, NYHA classes III and IV) than among the other patients. The least common reason for nonrecruitment was having a life expectancy <6 months (18/443). Age was a major factor for non-recruitment: 84% (187/223) of the nonrecruited patients with balance problems and 79% (149/189) of non-recruited patients with physical problems were 70 years old and older.

**Table 3:** Reasons for non-recruitment, based on the exclusion criteria and reasons for declining to participate in the HF-Wii study.

Reasons			n
**Nonrecruitment (n=443)^a,b^**			
	Balance problems	223	
	Physical problems	189	
	Inability to fill in the questionnaire	107	
	Cognitive impairment(s)	79	
	Visual impairment	52	
	Life expectancy <6 months	18	
**Declining to participate (n=723)^c,d,e^**			
	Not having time	93	
	Working or travelling a lot	82	
	Unwilling to come to follow up meetings	48	
	Having other illnesses	41	
	Already exercising a lot	41	

^a^Patients could be ineligible because of more than one criterion.

^b^Only the reasons given by at least 1 patient are reported.

^c^Multiple reasons for each patient were possible.

^d^Reasons were given by 569 patients (78.7%).

^e^Only reasons given by >30 patients are reported in the table.

Among the patients meeting the study criteria and invited to the HF-Wii study, 60.80% (723/1189) decided not to participate, and 79% percent (569/723) gave at least one reason for declining to participate (Table 3).

Among the reasons for declining, the 3 most often reported reasons were: “not having time,” “working or travelling a lot,” and “unwilling to come to follow-up meetings.” Not having time was the most common reason for declining participation in the HF-Wii study among patients 65-69 years of age and younger. It was more common among the youngest age group than among the other age groups to decline to participate in the HF-Wii study because they were already engaged in physical activities. Working or travelling a lot and not having time were the two most common reasons for declining to participate among patients 70 years and older.

## Discussion

Evaluations of digital health technologies are affected by selective nonparticipation that can prevent the representation of needs and interests of parts of the target population, bias research results, and generate conclusions that lead to inefficient solutions and new inequalities. The aim of this paper was to understand the predictors of participation in digital health research. For this purpose, an analysis of selective nonparticipation in a study evaluating an exergame technology for patients with HF (ie, the HF-Wii study) was conducted to examine whether and how (non)participation is associated with age, gender, and symptom severity. Selective nonparticipation was deconstructed and analyzed as resulting from two consecutive processes: (non)recruitment based on study requirements and self-selection as the individual decision.

Overall, results show that participants, compared with nonparticipants, had less severe HF symptoms and were younger, but did not differ by gender. The main reasons for nonrecruitment were balance or physical problems, whereas the main reasons for declining participation in the HF-Wii study were related to lack of time and other commitments (ie, working or travelling a lot).

More severe HF symptoms predicted the nonrecruitment of patients in our sample. Patients with such severity of symptoms often show balance problems or severe comorbidities that more often make them ineligible according to study criteria. This result agrees with previous findings on participation in health-related research showing that patients with poorer health status, such as frail patients [40], patients with cognitive impairment [41], and patients with poorer physical functioning [42], are less likely to be participants.

However, among the patients in our sample, HF symptom severity did not significantly affect self-selection. This contrasts with findings of previous research describing individuals who decline participation as being more likely to show worse health than participants [43-45]. One possible explanation is that the patients with more severe HF symptoms were identified as ineligible to participate in the HF-Wii study already in the initial screening phase because of a higher incidence of balance or physical problems and, thus, were not invited to participate in the study.

Age significantly predicted participation in the evaluation of the exergame intervention. Belonging to an older age group reduced the probability of participating through both the processes of (non)recruitment and self-selection. This confirms previous findings on participation in health-related research [46] and more specifically in both digital health [25,29] and HF research [47]. Such results are especially relevant in relation to the epidemiology of HF. As HF is more common among older people [48,49], it is crucial that the inclusion of older people in digital health research targeting HF is sustained.

Counter to expectations, patients 64 years old and younger were less likely to participate and more likely to be nonrecruited. This can be due to factors other than age and HF symptoms, which were, on average, not more severe than for other groups.

Gender did not affect the likelihood of participating. However, women represented less than one-third of the overall sample that entered the recruitment process and less than one-third of the participating group. This reflects difficulties in recruiting women with HF. Although the recruitment of women in HF studies has improved over time [50], the participation rate among women does still not reflect disease levels in the population [50,51]. In other studies, women were found to be more likely to decline the invitation to participate in digital health research [26,29]. This was not found for the sample in this study.

This study has some limitations. First, due to ethical clearance on collecting information about nonparticipants, only limited information on the patients was available. Such information did not include factors that can further explain participation in digital health research, such as level of education, digital skills, digital health literacy, and social participation. Future research should investigate the impact of such factors on both (non)recruitment and self-selection. On the other hand, it should be considered that collecting such detailed information, for example through a survey, might itself generate bias based on the nonresponse of some individuals. Therefore, although registered hospital information is limited, it can give an accurate description of selective non-participation. Second, for these analyses, reasons for refusal were only available grouped in main categories, which might have simplified the individual decision-making process for declining the invitation to participate in the HF-Wii study. Detailed reasons for declining the invitation to participate could have provided more insights into, for example, logistics-related and technology-related barriers to participation and allowed for a more elaborated description of the individual decision-making process. For example, in the full HF-Wii study it was found that 4% of the patients reported not wanting to participate because of the use of technology [32]. Future studies should further examine such reasons for refusal in combination with an analysis of the self-selection process. Third, as specific information on digital skills and technology-related aspects is not available, results from this study can also be relevant to the understanding of selective (non)participation in health research.

Selective nonparticipation in digital health research can prevent the production of results that appropriately inform research, policy, and practice on the impact of digital health technologies for the targeted populations. If participants and nonparticipants differ from one another, research results will not represent the target population of interest but rather a part of it. Groups that are often underrepresented in digital health research, such as people of older age and with poorer health, can be the most in need of accessing care and support [6] and can experience more barriers to using digital technologies [9,14]. Underrepresenting such groups implies overlooking their needs and interests, which are not necessarily expressed by their participating counterparts, and, as a result, miscalculating the impact of digital health technologies on the target population as a whole. Implementing digital health technologies that have been selectively evaluated might introduce further sources of exclusion and disadvantages to such groups with respect to their counterparts and contribute to widening old age inequalities.

We therefore recommend that a measure of selective nonparticipation is included in digital health research to identify overestimation and underestimation of the effects of digital health technologies due to the involvement of samples that do not reflect the target population. A measure of selective participation also allows researchers to employ further research strategies, such as focused recruitment of underrepresented groups or post-hoc adjustments of the results by weighting different groups depending on how well they are represented in the study sample.

## Abbreviations

AME: average marginal effect
FORTE: FORTE: Swedish Reserach Council for Health, Working Life and Welfare (Forskningsrådet för hälsa, arbetsliv och välfärd)
HF: heart failure
NYHA: New York Heart Association
OR: odds ratio
RCT: randomized controlled trial
